# PDE-5 inhibitors should be used post radical prostatectomy as erection function rehabilitation? Opinion: Yes

**DOI:** 10.1590/S1677-5538.IBJU.2017.03.03

**Published:** 2017

**Authors:** Laith M. Alzweri, Arthur L. Burnett

**Affiliations:** 1 The James Buchanan Brady Urological Institute and Department of Urology, The Johns Hopkins School of Medicine, Baltimore, MD, USA

**Keywords:** Phosphodiesterase 5 Inhibitors, Erectile Dysfunction, Penile Erection, Prostatectomy

## INTRODUCTION

Despite significant improvement in surgical techniques of radical prostatectomy (RP) since the nerve-sparing approach was introduced in 1982 (1), erectile dysfunction (ED) post RP remains a challenge for patients and urologists (2). Although most patients experience some degree of ED post RP, erectile function recovery rates vary according to age, baseline erectile function, comorbidities, and extent of nerve-sparing techniques (3, 4). The concept of penile rehabilitation has been evolving over the last two decades, in parallel with a better understanding of the basic scientific basis for ED post RP. In a recent systematic review of 11 randomized, controlled clinical trials (RCTs) on erection rehabilitation post RP in general, there was no significant improvement in spontaneous erectile function (unassisted by erection aids) rate of 20-25%. This rate was obtained from data in the control arm of trials after nerve sparing radical prostatectomy (NSRP) over the last two decades (5).

Phosphodiesterase phosphate-5 inhibitors (PDE5I) are the first line therapeutic option for organic ED, including ED post RP. In some studies, unassisted erectile function preoperatively was associated with preserved potency post RP in 94% of cases, but self-reported return to baseline erectile function status was shown in less than 40% and 23% of patients with and without the use of PDE5I, respectively (6).

The plausible hypothesis for the use of PDE5I in erection rehabilitation after RP has been investigated in multiple randomized, controlled trials. Although there is no consensus on the definition or algorithm for erection rehabilitation, a multidisciplinary approach addressing general and psychological well-being of patients and their partners, in addition to medical therapeutic options, would facilitate sexual function recovery post RP, including erectile function.

### ED post RP

ED post RP is mainly due to surgical trauma resulting in neurapraxia and/or injury of the cavernous nerve, which triggers a penile remodeling process. This process includes endothelial dysfunction and ischemic changes in the penile tissue with fibrosis of cavernosal smooth muscle. Different surgical approaches have been described to mitigate the risk of cavernous nerve injury (7).

### Definition of Erection Rehabilitation

Erection rehabilitation was introduced more than a decade ago, in response to an increasing understanding of the anatomical, pathophysiological and biochemical basis of ED post RP. It involves the use of any intervention or combination of interventions (i.e., medications and devices) with the goal of restoring erectile function to pretreatment levels, according to the fourth International Consultation of Sexual Medicine (ICSM 2015) (8).

### Evidence supporting PDE5I in ED post RP

PDE5I operate via a well-established mechanism of action for eliciting an improved erection response, through potentiating the NO-mediated relaxation of cavernosal smooth muscle by inhibiting the degradation of its downstream effector cyclic GMP (9). This mechanism of action pertains mainly to its therapeutic use in ED.

Evidence from animal models of cavernous nerve injury resembling the effects of ED post RP has supported the potential positive effect of PDE5I on erectile function recovery. PDE5I help alleviate penile remodeling in terms of promoting cavernous nerve regeneration, reducing inflammation and fibrosis of erectile tissues (10,11).

### Erection Rehabilitation RCTs

Multiple RCTs have attempted to study the use of PDE5I for erection rehabilitation post RP. Erectile function recovery in most RCTs was defined by (IIEF-Erectile function (EF) >21 or >22, and Sexual Encounter Profile question 3 [SEP3, successful intercourse rate]). The described “PDE5I-assissted erectile function recovery” during treatment period of the study, reflects ED post NSRP responsiveness to treatment with PDE5I.Erectile function recovery is reflected more accurately ,when IIEF-EF and SEP3 scores are reported based on spontaneous erections during the drug free washout period.

The first study which spearheaded this approach was done by Padma-Nathan et al. (12) in 2008: a randomized, double-blind and placebo-controlled trial for men with normal preoperative erectile function (combined score >7 in response to questions 3 and 4 of the International Index of Erectile Function questionnaire [IIEF]) treated with open NSRP. Nightly sildenafil administration for 36 weeks after surgery significantly increased the frequency of PDE5I-assissted erectile function (P=0.0156), with higher mean scores in response to questions 3 and 4 of the IIEF questionnaire. Four weeks after surgery all 125 men were randomized to double-blind sildenafil (50-100mg) or placebo nightly for 36 weeks, followed by a drug-free washout period for 8 weeks prior to erectile function assessment. Enrollment was stopped prematurely and only 76 men completed the trial, due to a suggested lack of treatment effect at an interim blinded analysis. In a post-termination analysis using Fisher exact test of men who completed the trial, spontaneous erectile function was reported by 27% and 4% of men who received sildenafil (50-100mg) and placebo, respectively. A longer duration of nocturnal erections was also reported in men who responded to sildenafil than non-responders (12).

In two double-blind RCTs [REINVENT (13) and REACTT (14)] Montorsi et al. assessed the effect of PDE5Is nightly versus on-demand on erection rehabilitation post NSRP. REINVENT (Recovery of Erections: Intervention with Vardenafil Early Nightly Therapy) in 2008 assessed the effect of nightly vardenafil (5 or 10mg) versus on-demand (5mg, 10mg or 20mg) taken beginning 4 weeks after NSRP and for 9 months, followed by a 2-month washout period. There was no significant improvement in terms of erectile function recovery (IIEF-EF >21, and [SEP3, successful intercourse rate]) after the washout period. On the other hand, there was a significant improvement of IIEF-EF scores in the on-demand users of vardenafil when compared with the placebo group (P=0.0001), 48.2% and 24.8%, respectively. SEP3 scores also improved significantly in on-demand users, when compared with nightly dosing or placebo. On-demand dosing of PDE5I is effective treatment in potentiating assisted erections post RP, with no clear positive effect on erection rehabilitation in the same setting (13).

In a large RCT (REACTT), Montorsi et al. (14) in 2014 assessed the effect of daily tadalafil (5mg ) versus on-demand (20mg) on erection rehabilitation taken beginning 4 weeks after NSRP and taken for 9 months, followed by a 6 week washout period, and subsequent 3 months open-label treatment with tadalafil 5mg once daily. Men with low and intermediate-risk prostate cancer, with good baseline erectile function preoperatively (IIEF-EF >22), and no medical conditions associated with ED underwent NSRP. ED was defined as [(patient-reported Residual Erection Function score ≤3: “penis is hard enough for penetration but not completely hard”)]. Outcomes measured were the rate of IIEF-EF score of >22, SEP3 score and stretched penile length.

The rate of achieving IIEF-EF score >22 was significantly higher (P=0.016) in the once-daily tadalafil (5mg) group, when compared to placebo (25.2% versus 14.2%) during the 9 months’ treatment period. There was no statistical significant difference between different groups in achieving IIEF-EF >22 after the washout period and open-label treatment. In a recent analysis, Mulhall et al (15) confirmed that changing the definition for erectile function recovery from IIEF-EF score ≥22 to the more strict definition of “returning back-to-baseline IIEF-EF” had no major impact. Tadalafil (5mg) used once-daily had no effect on erectile function recovery post NSRP, but it improved PDE5I-assisted erectile function after 9 months (15).

Although the change in stretched penile length was significantly less (P=0.032) in the on-demand group when compared to placebo, the difference of penile length loss was only 4.1mm. The suggested potential protective effect of early once-daily tadalafil (5mg) on cavernosal tissues after NSRP and prevention of penile length loss, was echoed in a 2015 analysis of the on-demand tadalafil use by Brock et al. (16).

A post open-label period analysis of the (REACTT) data (14) showed a significantly higher positive SEP3 response rate in the once-daily tadalafil (5mg) group than the placebo group during the double blinded treatment period (55.3% versus 37.8%, P=0.010). It was not significant when compared with the on-demand tadalafil (20mg) group. In a further analysis of the (REACTT) data (14) performed by Moncada et al. (17) in 2015, during the 9 months double-blind treatment , a statistically significant shorter time to PDE5I-assisted erectile function was associated with the once daily tadalafil (5.8 months = 0.03), and on-demand groups (9 months = 0.01), when compared to placebo (9.3 months).

Predictors for erectile function recovery post NSRP were investigated in a recent analysis of the (REACTT) (18). IIEF related predictors included: high preoperative sexual desire, satisfaction with sexual performance and confidence. Pertinently, once-daily tadalafil (5mg) was among the additional non-IIEF related predictors, including use of robotic surgery versus other approaches and quality of nerve sparing (18).

### Evidence synthesis

Although there is a rationale supporting erection rehabilitation post RP based on an increasing understanding of the postoperative changes in the physiology of erection and anatomical structures of the penis, there is no consensus to establish an algorithm for management of erection rehabilitation.

A clear distinction should be made between two endpoints, the recovery of erectile function post RP in terms of a patient’s ability to obtain an unassisted erection, and response to PDE5I treatment when used for organic ED post RP to obtain PDE5I-assisted erections. Erectile function recovery in most RCTs was based on self-reported functional parameters(IIEF-EF and SEP3 ),which reflects ED post NSRP responsiveness to PDE5I during treatment period of the study. Erectile function recovery is reflected more accurately, when IIEF-EF and SEP3 scores are reported based on spontaneous erections during the drug free washout period.

Evidence from animal models of cavernous nerve injury resembling the effects of ED post RP has supported the potential positive effect of PDE5I on erectile function recovery. PDE5I help alleviate penile remodeling in terms of promoting cavernous nerve regeneration and reducing inflammation and fibrosis of erectile tissues (10, 11). Nevertheless, multiple RCTs (12-14) did not show clear evidence of a sustained positive effect on spontaneous erectile function after using PDE5I for erection rehabilitation. The use of PDE5I whether on-demand or daily, was not shown to enhance patients’ ability to obtain an unassisted erection (i.e. erectile function recovery), at a statistically significant level after the washout period. There is also a potentially significant added health care cost with the daily use of PDE5I. Although tadalafil use was associated with a statistically significant effect in preserving stretched penile length after NSRP in two analyses, the difference in length was only 4.1mm (15, 16). It is unclear whether this improvement is clinically relevant.

## CONCLUSIONS

Our recent literature review supports the use of an on-demand approach of PDE5I to treat ED post NSRP by facilitating PDE5I-assisted erections. Despite evidence of positive effects of PDE5I on erectile tissue after cavernous nerve injury from animal models, in addition to penile length preserving effect in humans, there is no clear evidence to support a sustained positive effect of PDE5I use as erection rehabilitation post NSRP, in terms of a patients’ ability to obtain unassisted erections. It is possible that in the foreseeable future and with advancement at the basic science level, a better understanding would culminate in a rigorous and evidence based approach for erection rehabilitation post RP.

## References

[1] Walsh PC, Donker PJ (2017). Impotence Following Radical Prostatectomy: Insight into Etiology and Prevention. J Urol.

[2] Mulhall JP, Bivalacqua TJ, Becher EF (2013). Standard operating procedure for the preservation of erectile function outcomes after radical prostatectomy. J Sex Med.

[3] Tal R, Alphs HH, Krebs P, Nelson CJ, Mulhall JP (2009). Erectile function recovery rate after radical prostatectomy: a meta-analysis. J Sex Med.

[4] Ficarra V, Novara G, Ahlering TE, Costello A, Eastham JA, Graefen M (2012). Systematic review and meta-analysis of studies reporting potency rates after robot-assisted radical prostatectomy. Eur Urol.

[5] Schauer I, Keller E, Müller A, Madersbacher S (2015). Have rates of erectile dysfunction improved within the past 17 years after radical prostatectomy? A systematic analysis of the control arms of prospective randomized trials on penile rehabilitation. Andrology.

[6] Sopko NA, Burnett AL (2016). Erection rehabilitation following prostatectomy—current strategies and future directions. Nat Rev Urol.

[7] Salonia A, Burnett AL, Graefen M, Hatzimouratidis K, Montorsi F, Mulhall JP (2012). Prevention and management of postprostatectomy sexual dysfunctions part 2: recovery and preservation of erectile function, sexual desire, and orgasmic function. Eur Urol.

[8] Salonia A, Adaikan G, Buvat J, Carrier S, El-Meliegy A, Hatzimouratidis K (2017). Sexual Rehabilitation After Treatment For Prostate Cancer-Part 2: Recommendations From the Fourth International Consultation for Sexual Medicine (ICSM 2015). J Sex Med.

[9] Carson CC, Lue TF (2005). Phosphodiesterase type 5 inhibitors for erectile dysfunction. BJU Int.

[10] Sirad F, Hlaing S, Kovanecz I, Artaza JN, Garcia LA, Rajfer J (2011). Sildenafil promotes smooth muscle preservation and ameliorates fibrosis through modulation of extracellular matrix and tissue growth factor gene expression after bilateral cavernosal nerve resection in the rat. J Sex Med.

[11] Martínez-Salamanca JI, Zurita M, Costa C, Martínez-Salamanca E, Fernández A, Castela A (2016). Dual Strategy With Oral Phosphodiesterase Type 5 Inhibition and Intracavernosal Implantation of Mesenchymal Stem Cells Is Superior to Individual Approaches in the Recovery of Erectile and Cavernosal Functions After Cavernous Nerve Injury in Rats. J Sex Med.

[12] Padma-Nathan H, McCullough AR, Levine LA, Lipshultz LI, Siegel R, Montorsi F (2008). Randomized, double-blind, placebo-controlled study of postoperative nightly sildenafil citrate for the prevention of erectile dysfunction after bilateral nerve-sparing radical prostatectomy. Int J Impot Res.

[13] Montorsi F, Brock G, Lee J, Shapiro J, Van Poppel H, Graefen M (2008). Effect of nightly versus on-demand vardenafil on recovery of erectile function in men following bilateral nerve-sparing radical prostatectomy. Eur Urol.

[14] Montorsi F, Brock G, Stolzenburg JU, Mulhall J, Moncada I, Patel HR (2014). Effects of tadalafil treatment on erectile function recovery following bilateral nerve-sparing radical prostatectomy: a randomised placebo-controlled study (REACTT). Eur Urol.

[15] Mulhall JP, Brock G, Oelke M, Fode M, Probst KA, Henneges C (2016). Effects of Tadalafil Once-Daily or On-Demand vs Placebo on Return to Baseline Erectile Function After Bilateral Nerve-Sparing Radical Prostatectomy--Results from a Randomized Controlled Trial (REACTT). J Sex Med.

[16] Brock G, Montorsi F, Costa P, Shah N, Martinez-Jabaloyas JM, Hammerer P (2015). Effect of Tadalafil Once Daily on Penile Length Loss and Morning Erections in Patients After Bilateral Nerve-sparing Radical Prostatectomy: Results From a Randomized Controlled Trial. Urology.

[17] Moncada I, Bethencourt FR de, Lledó-García E, Romero-Otero J, Turbi C, Büttner H (2015). Effects of tadalafil once daily or on demand versus placebo on time to recovery of erectile function in patients after bilateral nerve-sparing radical prostatectomy. World J Urol.

[18] Montorsi F, Oelke M, Henneges C, Brock G, Salonia A, d’Anzeo G (2016). Exploratory Decision-Tree Modeling of Data from the Randomized REACTT Trial of Tadalafil Versus Placebo to Predict Recovery of Erectile Function After Bilateral Nerve-Sparing Radical Prostatectomy. Eur Urol.

